# Social health insurance and professional ethics in Romania: between legal framework and medical integrity

**DOI:** 10.3389/frhs.2026.1749779

**Published:** 2026-03-25

**Authors:** Roxana Elena Mirică, Ioana Soare

**Affiliations:** 1 Faculty of Medicine, University of Medicine and Pharmacy, Bucharest, Romania; 2 Department of Social Insurance Medicine, National Institute of Medical Expertise and Recovery of Work Capacity, Bucharest, Romania; 3 Gastroenterology Department, Regina Maria Private Healthcare Network, Bucharest, Romania; 4 Titu Maiorescu University, Bucharest, Romania

**Keywords:** healthcare system, medical practice, professional ethics, social health insurance, social insurance physician

## Abstract

**Background:**

Romania's social health insurance system is based on the Bismarck model and aims to provide universal healthcare access through compulsory contributions. While financing and institutional design have been widely studied, the ethical responsibilities of physicians, particularly social insurance physicians, remain under-explored.

**Objective:**

This study analyzes Romania's social health insurance system through an integrated institutional and ethical lens, focusing on social insurance physicians who operate at the interface of clinical assessment and social protection eligibility.

**Methods:**

A structured narrative review was conducted, synthesizing national legislation, international ethical frameworks (Declaration of Geneva, International Code of Medical Ethics), peer-reviewed literature (2010–2025), and comparative analyses with European health insurance models. The analysis identified systemic challenges, including underfunding, workforce migration, and demographic pressures, and examined their impact on professional ethics.

**Results:**

Romania ensures broad formal coverage, yet persistent challenges—underfunding, regional disparities, informal payments, and physician shortages—create ethical tensions for social insurance physicians. Comparative analysis shows that other European countries (Germany, France, Netherlands) mitigate such pressures through stronger institutional safeguards, higher funding, and protected professional autonomy.

**Conclusion:**

This study contributes to health services research by conceptualizing ethical vulnerability in resource-constrained insurance systems and linking institutional structures to professional autonomy, fairness, and system resilience. Findings highlight the need to strengthen ethical frameworks, safeguard physician independence, and align institutional reforms with ethical governance to ensure transparent, equitable, and resilient social health insurance systems.

## 1 Background

Health systems worldwide aim to reconcile three key objectives: accessibility, efficiency, and equity. Two predominant models are typically distinguished. The Bismarck model (named after the Prussian Chancellor Otto von Bismarck), developed in 19th-century Germany, relies on compulsory contributions from employees and employers, with services delivered by both public and private providers. The Beveridge model (Named after William Beveridge, the daring social reformer who designed Britain's National Health Service), by contrast, is financed through taxation and characterized by a state-run structure, as seen in the United Kingdom and Scandinavia.

Romania has formally adopted the Bismarck model since the reform of 1997, protecting the whole population of the country such as wage earners, the pensioners, even if the unemployed, but having the obligation to assure their health according to the law (1) and which created the National Health Insurance House (CNAS) and a network of county insurance houses that function through the funds. This reform shifted the financing of health from general state budgets to earmarked social contributions, in line with European practice (2).

Yet financing mechanisms alone do not ensure quality or fairness in healthcare delivery. Physicians, as gatekeepers of both medical knowledge and public trust, are guided by professional ethics (3), commitments, responsabilities and highest standards which guide doctors in fulfilling their obligation to provide competent medical care to the patients (4). The World Medical Association (WMA) has articulated international ethical frameworks, including the Declaration of Geneva (2017revision)and the International Code of Medical Ethics(2022), which emphasize dignity, fairness, and integrity (5). In Romania, the College of Physicians oversees the medical profession and enforces national ethical codes (6).

This paper addresses the intersection between Romania's social health insurance system and professional medical ethics, with particular emphasis on the responsibilities of social insurance physicians.

### 1.1 Scientific contribution and theoretical gap

Although the Romanian social health insurance system has been examined from economic, legal, and policy perspectives, limited research has explored the relationship between institutional design and professional medical ethics within social insurance frameworks. In particular, the ethical role of social insurance physicians—who function at the interface between clinical assessment and eligibility determination for social benefits—remains under-theorized in health systems research.

This study addresses this gap by developing an integrated institutional-ethical perspective on Romania's Bismarck-type health insurance model. Rather than analyzing financing and governance mechanisms in isolation, the paper examines how structural characteristics of the system—such as underfunding, demographic pressure, and physician migration—interact with professional ethical obligations.

The article makes three main contributions. First, it conceptualizes social insurance physicians as key actors in ensuring fairness, transparency, and institutional trust within contributory health systems. Second, it introduces the concept of ethical vulnerability in resource-constrained insurance systems, highlighting how systemic pressures may affect professional independence and objectivity. Third, through comparative reference to selected European models, it situates the Romanian case within broader debates on health system resilience and ethical governance.

By linking institutional arrangements with ethical accountability, this study contributes to health services research by framing professional ethics not only as an individual duty but as a structural determinant of equity and legitimacy in social health insurance systems.

## 2 The social health insurance system in Romania

### 2.1 Historical background

The Romanian healthcare system operated predominantly as a state-funded Beveridge-type model during the communist era, with limited patient choice and centralized management. The reform of 1997 introduced the Bismarck-inspired system, aimed at increasing financial sustainability and aligning Romania with European partners (7).

### 2.2 Institutional framework

**Figure 1:**
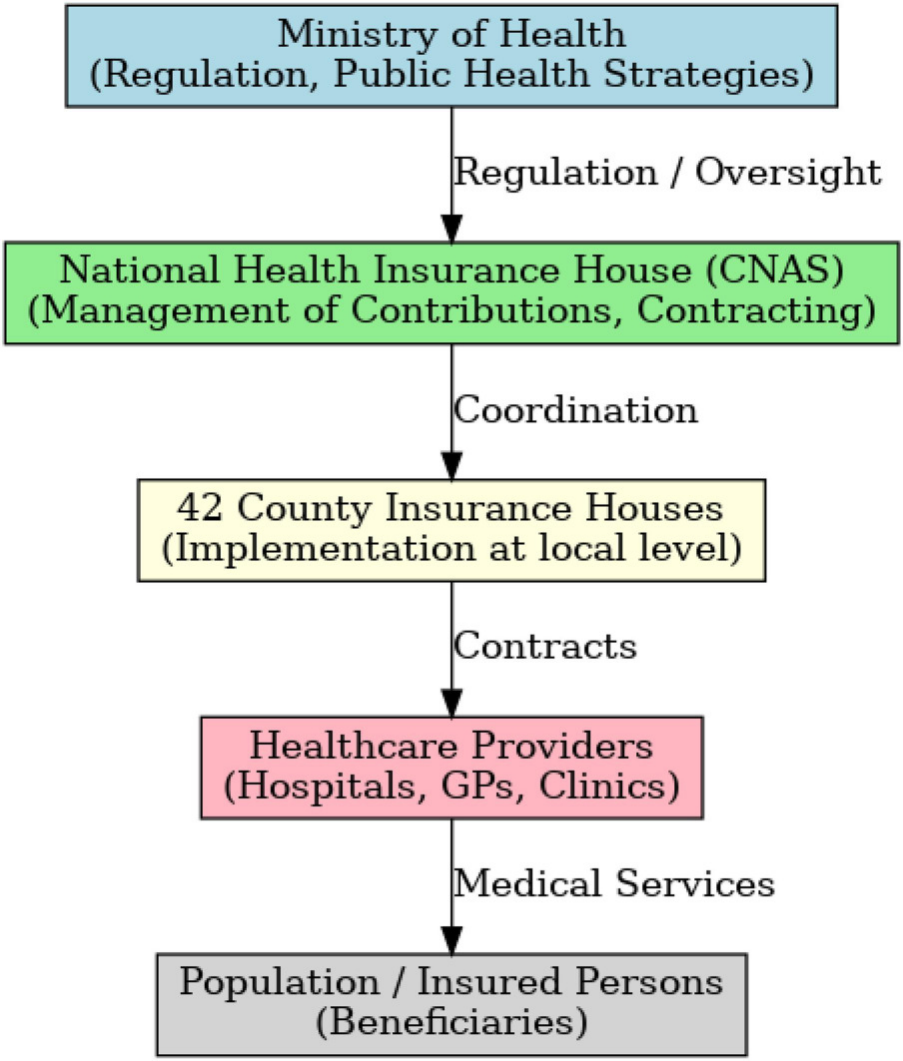
Organizational structure of the Romanian social health insurance system.

The National Health Insurance House (CNAS) acts as the central body responsible for managing contributions and contracting healthcare services. It cooperates with 42 county-level insurance houses. The Ministry of Health maintains regulatory authority, particularly regarding public health strategies, while the College of Physicians supervises the medical profession (Figure 1).

### 2.3 Financing and coverage

Healthcare is funded mainly through payroll contributions (currently 10% of gross income).The state subsidizes contributions for vulnerable groups such as pensioners, children, and the unemployed. In theory, the system ensures universal coverage, yet disparities remain in practice.

According to the Organization for Economic Co-operation and Development—OECD (2022) (8),Romania spends approximately5.7% of Gross Domestic Product (GDP)on healthcare, compared to an EU average of nearly10%.Out-of-pocket payments account for nearly 20% of total health expenditure, creating barriers to equitable access (9).

### 2.4 Challenges in access

Despite universal coverage on paper, rural populations face shortages of medical staff and facilities. Informal payments persist, undermining equity and transparency. Waiting times and uneven distribution of resources remain major systemic weaknesses.

## 3 Methods

### 3.1 Study design

This study was conducted as a structured narrative review, aiming to explore the intersection between Romania's social health insurance system and professional medical ethics. A narrative review was selected because it allows integration of diverse sources, including legislation, ethical codes, institutional reports, and comparative health system analyses, which are not suitable for quantitative synthesis. This approach enables the development of a conceptual framework linking institutional structures to professional ethical responsibilities.

### 3.2 Data sources

We conducted a comprehensive search of peer-reviewed literature and official documents using the following databases and sources:

- PubMed, Scopus, and Web of Science for academic publications;

Reports and statistical data from the Organization for Economic Co-operation and Development (OECD), World Health Organization (WHO), and Romanian national institutions (National Health Insurance House, Ministry of Health);

- International ethical frameworks, including the Declaration of Geneva (2017 revision) and the International Code of Medical Ethics (2022);

National legislation and regulatory documents, including the Code of Medical Deontology adopted by the Romanian College of Physicians.

In addition to national and legislative sources, recent literature on health system performance, sustainability, and ethical considerations in healthcare governance was systematically examined to provide up-to-date conceptual grouding for the review. Robust social protection programs enable governments to improve citizens’ access to healthcare and resources, leading to better health outcomes. These results highlight the importance of prioritizing social protection policies to reduce health disparities and strengthen public health across the EU (10).

### 3.3 Inclusion and exclusion criteria

Inclusion criteria:

- Peer-reviewed articles, reviews, and comparative studies on health insurance systems, professional ethics, and social insurance physicians;
- Official reports, legislative documents, and policy briefs relevant to Romania or comparative European contexts;
- Publications between 2010 and 2025, with emphasis on recent studies from 2021 to 2025.

Exclusion criteria:

- Commentaries, editorials, or non-peer-reviewed opinion pieces;
- Studies not relevant to European social health insurance systems;
- Literature without explicit reference to ethical, institutional, or policy frameworks.

### 3.4 Data extraction and analysis

Selected documents were systematically reviewed to identify key themes related to:

- Institutional characteristics of the social health insurance system in Romania;
- Ethical principles guiding professional conduct, particularly of social insurance physicians;
- Comparative insights from other European Bismarck-type or hybrid health insurance models.

Information was extracted using a thematic coding approach, categorizing data under institutional structure, financing, workforce, ethical challenges, and policy implications. Comparative analysis was then performed to identify gaps, strengths, and transferable lessons for Romania's health system.

### 3.5 Professional ethics in medicine

The medical profession is guided by universally recognized ethical principles, including beneficence (promoting patient well-being), non-maleficence (avoiding harm), respect for confidentiality, objectivity (without bias or discrimination), and independent judgment (avoiding conflicts of interest) (4, 11).

International guidelines: The Declaration of Geneva commits physicians to prioritize patient health, uphold confidentiality, and practice with integrity, while the International Code of Medical Ethics (2022) details responsibilities toward patients, colleagues, society, and the profession (5).

National framework: In Romania, the Code of Medical Deontology adopted by the College of Physicians aligns with international norms, regulating confidentiality, informed consent, fairness, and integrity. The Ministry of Health ensures integration of ethical standards into professional regulations and institutional oversight.

### 3.6 Ethical considerations

As this study relied solely on publicly available documents and literature, no primary data collection involving human participants was performed. All analyses were conducted in accordance with ethical standards for research using publicly available data.

## 4 Results

### 4.1 The broader framework of social insurance in Romania

The Romanian social protection system is structured around several branches of social insurance, designed to safeguard citizens against major social risks. Two main components can be distinguished: social health insurance and pension insurance.

The Social Health Insurance System, coordinated by the National Health Insurance House (CNAS)and county-level insurance houses, provides access to medical services for insured persons, financed through mandatory health contributions. This branch is directly linked to the provision of medical care and is supervised by the Ministry of Health, which ensures regulatory and strategic oversight.

**Figure 2:**
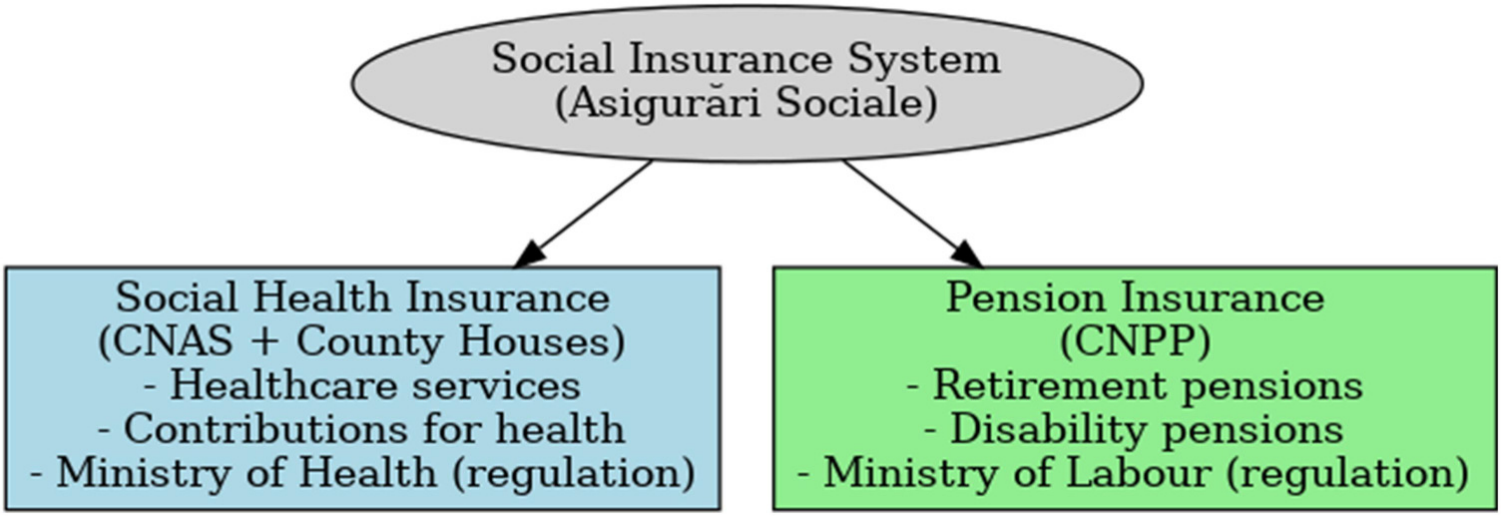
The institutional framework of social insurance in Romania, highlighting the distinction between the health insurance and pension insurance systems.

In parallel, the Pension Insurance System is administered by the National House of Public Pensions (CNPP), under the authority of the Ministry of Labour and Social Solidarity. It covers retirement pensions, disability pensions, and other social benefits context, medical expertise also plays a crucial role, as physicians are required to evaluate work capacity, disability, and eligibility for certain social benefits (Figure 2).

Therefore, while this article focuses primarily on the ethical dimensions of the medical profession within the social health insurance system, it is important to acknowledge that social insurance physicians may operate at the intersection of both systems. Their evaluations influence not only access to healthcare services but also eligibility for pensions and social benefits. This dual responsibility underscores the need for a robust ethical framework, which will be further explored in the following section.

## 5 Discussion

### 5.1 Interpreting the findings

Our analysis shows that while Romania's social health insurance system provides broad formal coverage, systemic challenges such as underfunding, workforce shortages, informal payments, and regional disparities persist (8, 9). These challenges are closely linked to institutional design, demographic pressures, and the migration of medical professionals. For example, physician emigration reduces the pool of qualified social insurance physicians, increasing workload and potentially affecting the objectivity of medical assessments. Social insurance physicians operate at the intersection of healthcare provision and social protection eligibility. This dual role generates ethical tensions between fulfilling legal obligations, maintaining professional independence, and ensuring equitable treatment of claimants. These tensions illustrate the concept of ethical vulnerability, where structural constraints—rather than individual failings—can compromise professional ethics (12–15).

### 5.2 Theoretical implications

Our findings confirm and extend theoretical frameworks on professional autonomy and institutional trust (3, 4). In Bismarck-type systems, physician independence is crucial for legitimacy and fairness, yet in resource-constrained contexts like Romania, systemic pressures challenge this autonomy (12, 13). By framing ethical vulnerability as a structural concern, rather than purely individual, this study contributes a conceptual perspective linking institutional features to ethical behavior.

Furthermore, the results support the notion that ethical standards are not only a personal duty but a structural determinant of system resilience and equity (5, 11). This aligns with comparative literature from Germany and France, where higher funding, pluralistic insurance models, and strong institutional safeguards reinforce both professional autonomy and public trust (8, 11).

### 5.3 Comparative insights

Comparative analysis indicates that Romania shares common challenges with other European countries but experiences amplified effects due to limited resources (8, 9).

- Germany: Strong physician autonomy and pluralistic insurance funds buffer ethical pressures (8).
- France: High state oversight ensures access while supporting professional independence (11).
- Netherlands: Regulated competition and universal acceptance reinforce fairness and efficiency (8).

Romania, by contrast, faces higher out-of-pocket costs, uneven access, and informal practices, which exacerbate ethical dilemmas for social insurance physicians (9, 15). These comparisons highlight potential best practices, such as safeguarding physician independence, improving transparency, and aligning financial incentives with ethical governance.

Recent OECD analyses confirm persistent inequities in access to care across the Romanian health system, with unmet healthcare needs remaining high particularly among vulnerable populations and rural residents, underscoring structural barriers that have ethical implications for equity and fairness in service delivery (16).

Linking premiums to behavior may undermine solidarity in health insurance, highlighting the need to consider broader issues such as big tech's role and the health economy interplay (17).

### 5.4 Policy and practical implications

The study emphasizes several actionable points:

1. Strengthen institutional safeguards for social insurance physicians to protect independent decision-making (12, 13).
2. Increase funding and resource allocation, particularly in rural areas, to reduce workload and ethical pressures (8, 9).
3. Enhance transparency and communication with claimants to build public trust (13, 14).
4. Integrate ethical considerations into digital transformation, such as AI-based assessments, ensuring privacy, fairness, and accountability (13). Recent studies on the ethical and social implications of digital technologies and AI in healthcare highlight emerging concerns about fairness, transparency, bias, and professional autonomy, which have important parallels with ethical challenges, faced by social insurance physicians in technology –driven assessment environments. Major gaps remain regarding the ethics of AI in healthcare, particularly in result disclosure, legal liability, and acceptance by patients and clinicians.Future studies should address these issues to ensure responsible and effective AI implementation in medical practice (18).

### 5.5 Limitations and future directions

While this narrative review integrates legislative, ethical, and comparative evidence, it does not include primary data collection. Future research could examine practitioner experiences and quantitative outcomes of ethical frameworks in social insurance practice (12, 13). Additionally, longitudinal studies tracking reforms and migration trends could strengthen understanding of systemic ethical dynamics (15).

### 5.6 Summary

In sum, Romania's social health insurance system demonstrates that professional ethics and institutional design are deeply intertwined. By conceptualizing ethical vulnerability and linking structural constraints to physician behavior, this study provides a framework for strengthening fairness, transparency, and resilience in social insurance systems (3–5, 12, 13, 15).

## 6 Conclusion

The Romanian social health insurance system, rooted in the Bismarck model, provides formal universal coverage but faces persistent challenges, including underfunding, workforce shortages, and uneven access. Within this context, social insurance physicians play a pivotal role, operating at the intersection of clinical assessment and social protection eligibility. Their adherence to professional ethics is essential for ensuring fairness, transparency, and public trust.

This study contributes to health services research by:

1. Conceptualizing the ethical vulnerability of social insurance physicians in resource-constrained systems;
2. Integrating institutional and ethical perspectives to highlight how system design affects professional autonomy and equity;
3. Providing a comparative European perspective, identifying both structural gaps and transferable best practices.

By linking professional ethics to institutional resilience, the findings underscore that ethical governance is not merely an individual duty but a structural determinant of system legitimacy. Strengthening ethical frameworks, improving institutional safeguards, and addressing systemic pressures are critical steps toward building a fairer, more transparent, and resilient social health insurance system in Romania and similar contexts.

## References

[1] Viorela-Ligia V, Ioan N. The social health insurances in Romania – realities and perspectives; Văidean Viorela-Ligia, Nistor Ioan; (2010) Available online at: https://doctorat.ubbcluj.ro/sustinerea_publica/rezumate/2010/finante/Vaidean_Viorela_EN.pdf (Accessed March 13, 2026).

[2] Mossialos E Dixon A Figueras J Kutzin J. FundingHealthCare: Options for Europe. Maidenhead: Open University Press (2019).

[3] AMA Principles of Medical Ethics. Adopted June1957; revised June1980; revised June 2001.

[4] Canadian Medical Association Code of Ethics and Professionalism. (2018).

[5] World Medical Association International Code of Medical Ethic. (2022).

[6] The Code of Medical Ethics of the Romanian College of Physycians. Lawno.306 of 28 June 2004.

[7] Vlădescu C Scîntee SG Olsavszky V Hernández-Quevedo C Sagan A. Romania: health system review. Health Syst Transit. (2016) 18(4):1–170. 10.1787/9789264265882-en 27603897

[8] OECD. Health at a Glance: Europe2022. Paris: OECD Publishing (2022).

[9] World Health Organization. Global Health Expenditure Database. Geneva: WHO (2021).

[10] Bocean CG Varzaru AA. Assessing social protection influence on health status in the European union. Front Public Health. (2024) 12:1287608. 10.3389/fpubh.2024.1287608 38528863 PMC10962762

[11] Beauchamp T Childress J. Principles of Biomedical Ethics(8th ed.). Oxford: Oxford University Press (2019).

[12] European Union of Medicine in Assurance and Social Security (EUMASS). Guidelines of Conduct for Inurance Physicians –European Union of Medicine in Assurance and Social Security

[13] Legea95, legea medicului, Mircea Boulescu Marcel Ghita. Editura didactica si pedagogica. (2001).

[14] World Medical Association. International Code of Medical Ethics. Geneva: World Medical Association (2022).

[15] OECD. Recent Trends in International Migration of Doctors and Nurses. Paris: OECD Publishing (2020).

[16] OECD. Access and Quality of Care in Romania’s Healthcare System. Paris: OECD Reviews of Health Systems: Romania 2025. OECD Publishing (2025).

[17] Bredthauer CJ Kuhn I Buyx A. The ethics of behaviour-based insurance models: solidarity-based concerns in Germany’s statutory health insurance. Health Policy. (2025) 156:105318. 10.1016/j.healthpol.2025.105318 40250334

[18] Wang Y Federman A Wurtz H Manchester MA Morgado L Scipion CEA The evolving literature on the ethics of artificial intelligence for healthcare: a PRISMA scoping review. Front Digit Health. (2025) 3:1701419. 10.3389/fdgth.2025.1701419 PMC12675450 41357434

